# Eco-friendly fabrication of vanadium nanoparticles *via Fusarium solani* with dual antifungal and anticancer bioactivities

**DOI:** 10.1039/d5ra08636a

**Published:** 2026-01-08

**Authors:** Saman M. Mohammed, Haider M. Hamzah

**Affiliations:** a Department of Biology, College of Education, University of Sulaimani Sulaymaniyah 46001 Iraq saman.mohammed@univsul.edu.iq; b Department of Biology, College of Science, University of Sulaimani Sulaymaniyah 46001 Iraq haider.hamzah@univsul.edu.iq

## Abstract

Nanotechnology is driving significant advancements across medicine, environmental science, and materials engineering. However, conventional nanoparticle synthesis often uses toxic reagents and energy-intensive protocols, raising environmental sustainability concerns. We developed a green, mycosynthesis approach for vanadium nanoparticles (VNPs), employing *Fusarium solani* as a biological reducing and stabilizing agent. The tolerance index of *F. solani* was determined using different precursor concentrations. Synthesis was optimized using pH (5.0, 7.0, and 9.0) and temperature (25 °C, 30 °C, and 35 °C). The optimized VNPs were characterized and evaluated as antifungal and antibiofilm agents against clinical *Candida albicans* strains and as anticancer agents against pancreatic cell lines. Optimization revealed that nanoparticle synthesis was most efficient with antifungal activity at pH 7.0 and 30 °C, as indicated by surface plasmon resonance (SPR) peaks around 370 nm. Fourier-transform infrared (FTIR) spectroscopy identified key functional groups including hydroxyl, amide, and carboxylate derived from fungal biomolecules, suggesting their role as natural capping and stabilizing agents. X-ray diffraction (XRD) confirmed highly pure, crystalline vanadium oxide nanoparticles exhibiting a rhombohedral V_2_O_3_ phase. VNPs displayed significant antimicrobial and biofilm disruption activity against *C. albicans* and strong anticancer potential against T3M-4 and CD18/HPAF pancreatic cancer cell lines, with IC_50_ ∼ 4 µg mL^−1^. These results underscore fungal-mediated synthesis as a viable approach for generating multifunctional, biocompatible nanomaterials for antifungal and anticancer therapy.

## Abstract 2

Green synthesis of VNP using *F. solani* demonstrates potent dual bioactivity against fungal pathogen and cancer cells, offering a sustainable approach to multifunctional nanomedicine.

## Graphical abstract

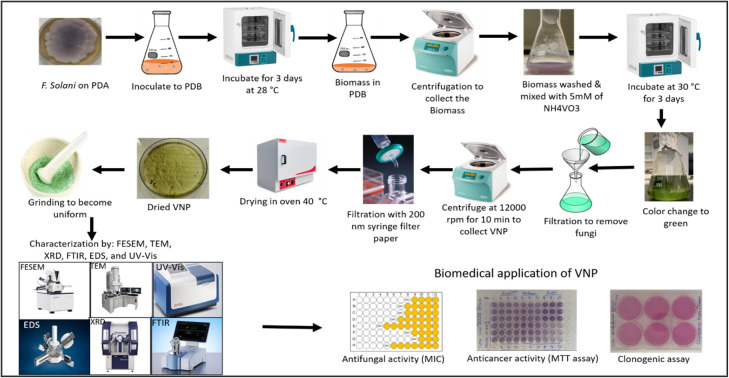

## 1 Introduction

Antimicrobial resistance (AMR) is a growing international health emergency that poses a challenge to the successful prevention and therapy of a myriad of infections caused by fungi and bacteria. Globally, AMR is responsible for approximately 1.27 million deaths each year, and by 2050 could become a top cause of death, surpassing cancer if left uncontrolled.^1^ Fungal infections, especially those caused by opportunistic species such as *Candida* species, represent an important and increasing worldwide health issue, particularly in immunocompromised hosts. The therapeutic repertoire, including azoles, echinocandins, polyenes, and combination regimens, has proven inadequate due to the appearance of resistant fungal forms.^2^ Resistance mechanisms involve gene alterations in drug target enzymes, overexpression of efflux transporters, biofilm-mediated protection, and adaptation to stress responses.^3,4^ The limited innovation of new antifungal agents and the increasing prevalence of invasive fungal infections with high mortality rates strongly underscore the need for alternative antifungals with varied mechanisms of action.

Beyond infectious diseases, similar challenges of resistance and limited therapeutic options plague cancer treatment. Cancer is among the most urgent international health issues and caused almost 10 million deaths in 2020. Traditional cancer treatments suffer from poor selectivity, harsh side effects, and the emergence of drug-resistant tumor cells,^5^ with pancreatic cancer exhibiting one of the lowest survival rates.^6^ These problems necessitate the investigation of new modalities capable of achieving higher selectivity, reduced toxicity, and improved therapeutic ratios. In this context, nanotechnology represents a promising approach to improve targeting, and circumvent mechanisms of resistance for antimicrobial and antifungal applications,^7–9^ nanoparticles hold promise through reactive oxygen species (ROS) generation, microbial membrane disruption, anti-biofilm activities, and intracellular interactions. These features enable nanoparticles to bypass conventional resistance mechanisms by simultaneously targeting multiple cellular pathways.^10,11^ Green or biological synthesis of nanoparticles using plants, bacteria, and fungi^12–15^ is emerging as a preferred approach compared to chemical and physical methods because these organisms secrete extracellular reductive enzymes, have high tolerance to heavy metals, and produce sufficient amounts of biomass.^16–18^ Developing safe and broad-spectrum therapeutics remains a major scientific challenge, *Fusarium* species show great potential in this regard, as their biosynthesized nanoparticles, such as silver nanoparticles (AgNPs), have demonstrated cytotoxic activity against mammalian cell lines, including the HeLa cervical cancer line.^19^

In recent years, mycosynthesis of NPs has been developed, and among fungi, *Fusarium solani* is recognized to have the enzymes that serve as both reducing and capping agents.^20–22^ Research studies revealed that silver, copper, and zinc nanoparticles were synthesized using *F. solani* extracellularly through the reduction of their respective metal ions *via* the secretion of extracellular chemical substances; these enzymes, proteins, and aromatic compounds function as electron shuttles to promote metal ion reduction and as stabilizing agents for the newly synthesized nanoparticles.^23–25^ The mycosynthesis of NPs is beneficial in many ways, such as being eco-friendly, less toxic, cost effective, and producing biocompatible nanomaterials.^26^ Mycosynthesis methods for NP synthesis provide dual advantages of sustainability and medical efficacy, serving as a significant platform for the production of silver, gold, and zinc oxide nanoparticles with antifungal, antibiofilm, and anticancer attributes,^27–29^ with recent advancements encompassing the generation of vanadium oxide nanoparticles.

Vanadium is a transition metal with different oxidation states that is exceptionally effective in catalysis and redox reactions. Vanadium is most often found in oxides with the following oxidation states: +5 (V_2_O_5_), +4 (VO_2_), +3 (V_2_O_3_), and +2 (VO).^30,31^ Vanadium oxide nanoparticles in nanoscale form show higher reactivity, a larger surface area, and a greater oxidative capacity, which makes them good candidates for use in antimicrobial and cancer treatment therapies. Vanadium-based nanoparticles have antimicrobial activity due to the generation of ROS, that elevates oxidative stress to cause lipid peroxidation, protein denaturation, and DNA breaks in microbial cells.^32,33^ VNPs disrupt essential enzymes and inhibit energy production and signal transduction pathways.^34,35^ This multi-targeted mechanism of action lowers the risk of resistance development compared to traditional single-target antimicrobials.^36^ Importantly, vanadium compounds can be toxic at high levels, but nanoscale formulations have shown better biocompatibility profiles due to controlled release kinetics and reduced systemic exposure.^37–40^ Studies have shown that appropriately sized VNPs exhibit selective toxicity toward cancer and microbial cells while maintaining acceptable safety margins in normal cells,^41,42^ making them viable candidates for therapeutic development.

In the context of mycosynthesis, *F. oxysporum* has been utilized to biosynthesize VNPs, which have shown dose-dependent antifungal activity, including inhibition of mycelial growth and suppression of spore germination, as well as cytotoxicity against MCF-7 breast cancer cells.^43^ Furthermore, plant-extract-derived VNPs have demonstrated both antioxidant and anticancer properties, including activity against colorectal cancer cell lines.^44,45^ Among fungal species, notably *F. oxysporum* and *Ganoderma lucidum* extracts have been used for the synthesis of VNPs,^43,46^ with effective nanoparticle formation and biological activity. However, the use of fungal biomass from *F. solani* remains limited in this domain. Moreover, systematic optimization studies involving pH variation and synthesis temperature have been scarcely explored, leaving critical gaps in understanding how these parameters influence antimicrobial and anticancer efficacy.

The present study is the first trial to mycosynthesis V_2_O_3_ and optimization of VNPs using *F. solani* biomass and evaluates their antifungal and biofilm disruption activities against *C. albicans* and anticancer activity against two common pancreatic cancer cell lines. This integrative approach aims to contribute to the development of novel, biologically synthesized VNPs for combating multidrug-resistant pathogens and cancer.

## 2 Materials and methods

### 2.1 Materials

Ammonium metavanadate and crystal violet were purchased form Biochem Chemopharma (France), flat bottom cell culture plates from Sorfa life science, China. Sabouraud dextrose broth (SDB), potato dextrose agar, and potato dextrose broth were from Liofilchem, (Italy). Sodium hydroxide and hydrochloric acid were from Sigma-Aldrich (Germany). Ethanol purchased from Merck (Germany), Dulbecco's Modified Eagle Medium (DMEM), fetal bovine serum, l-glutamine and penicillin streptomycin from Fisher Scientific (USA).

### 2.2 Fungal strain and biomass preparation

*Fusarium solani* was isolated from the sediment of the Tanjaro River and identified based on morphological features and internal transcribed spacer (ITS) rDNA sequencing in a previous study.^18^ The fungus was routinely cultured on potato dextrose agar (PDA) plates at 28 °C. For nanoparticle synthesis, the fungus was grown in potato dextrose broth (PDB) in a shaker incubator at 28 °C for 3 days. Following incubation, the fungal biomass was separated from the culture medium by centrifugation at 5000 rpm for 20 min, followed by washing with sterile distilled water to remove media residues. The biomass was then weighed and prepared for nanoparticle synthesis.

### 2.3 Vanadium tolerance analysis

The vanadium tolerance of *F. solani* was assessed using a standardized conidial suspension from a potato dextrose broth (PDB) culture using sterile gauze. In the agar-based vanadium tolerance assay, the growth response of the organism to different concentrations of ammonium metavanadate (NH_4_VO_3_) was assessed. The fungal conidial concentration of 1 × 10^5^ conidia mL^−1^ was adjusted after collection and counting. Potato dextrose agar (PDA) was prepared and cooled to 50 °C, then amended with filter-sterilized NH_4_VO_3_ at concentrations of 1, 2, 3, 4, 5, and 10 mM, and poured into Petri dishes. A plate without vanadium treatment was included as a negative control.^47^ Plates were inoculated centrally with 50 µL of the conidial suspension, sealed, and incubated at 28 ± 1 °C in the dark for 72 h. Fungal growth was assessed by measuring colony diameter. The Tolerance Index (TI) was calculated, with values approaching 1 indicative of greater metal resistance.^48^ Colony diameter measurements were performed using ImageJ version 1.54g.

### 2.4 Biosynthesis of vanadium nanoparticles

Vanadium nanoparticles (VNPs) were synthesized according to the method of Hamzah *et al.*,^19^ in 2018 with slight modifications. A vanadium precursor solution was prepared by dissolving ammonium metavanadate (NH_4_VO_3_, molecular weight 116.9 g mol^−1^) in 100 mL of sterile distilled water to obtain a 5 mM concentration. The fungal biomass was added to the solution in a 1 : 10 (w/v) ratio and incubated under static conditions at ambient temperature. The reaction mixture was maintained until a noticeable color shift from pale pink to dark green indicated nanoparticle formation. Subsequently, the mixture was centrifuged at 12 000 rpm for 15 min to collect the VNPs, washed three times with distilled water, and filtered in succession using Whatman no. 1 filter paper and a Millipore filter (0.22 µm). An oven was used to dry the purified VNPs at 40 °C. The VNPs were then resuspended in distilled water, and characterized by UV-vis spectroscopy.

### 2.5 Initial antimicrobial screening

The myco-synthesized VNPs (at neutral pH and 28 °C) were tested for antimicrobial activity against clinical *Candida albicans* isolate (0.5 McFarland standard, 1 × 10^6^ CFU mL^−1^) using the disc diffusion assay. The blank discs were loaded with 100 µL of VNPs and placed on Mueller Hinton agar, while fungal extract and vanadate solution were used as controls. After incubation at 35 °C for 24 h, the zones of inhibition (ZOI) were determined.

### 2.6 Optimization of synthesis temperature and pH

To determine the ideal temperature for the synthesis, the biosynthesis process was carried out at 25 °C, 30 °C, and 35 °C, while maintaining the pH at 7.0.^49^ UV-vis spectroscopy was used to characterize the VNPs, and the disc diffusion assay was used to assess their effectiveness against *C. albicans*. The purpose of this step was to identify the optimum temperature conditions for synthesizing nanoparticles with the highest antimicrobial efficacy. Following temperature optimization, the effect of pH on nanoparticle synthesis was examined. The reaction medium was adjusted to pH 5.0 (acidic), pH 7.0 (neutral), and pH 9.0 (alkaline) using dilute HCl or NaOH prior to synthesis.^50^ VNPs synthesized at each pH were isolated, purified, characterized by UV-vis spectroscopy, and tested for antifungal activity using the disc diffusion assay. Inhibition zones diameters were measured to evaluate the influence of pH and temperature on nanoparticle antifungal activity.

### 2.7 Vanadium nanoparticles characterization

The colloidal solution of mycosynthesized VNPs was characterized using a variety of physicochemical analyses to ensure the formation, crystallinity, size, shape, and surface chemistry. The following instruments and methods were employed:

UV-visible spectroscopy (PerkinElmer, Lambda 365, USA) was used to monitor optical properties of VNPs. X-ray diffraction (Phillips, PW1730, The Netherlands) was performed to identify diffraction peaks corresponding to crystalline phases of vanadium oxides. Fourier-transform infrared spectroscopy (PerkinElmer 2, USA) was conducted to identify functional groups derived from fungal metabolites, suggesting their role in reduction and capping of the nanoparticles. Field emission scanning electron microscopy (FESEM) (Tescan, MIRA3, Czech Republic) and transmission electron microscopy (TEM) (Phillips, CM120, The Netherlands) were used to visualize nanoparticle morphology. The images revealed predominantly irregular nanoparticles with an average size in the nanometer range. Energy-dispersive X-ray spectroscopy (Tescan, MIRA3, Czech Republic) was performed to confirm the presence of vanadium as the principal element, along with minor biomolecular residues. The zeta potential was measured using a Zetasizer (Horiba, SZ100, Japan) to assess the surface charge of the VNP suspension.

### 2.8 Antimicrobial activity

The antimicrobial activity of biosynthesized VNPs was assessed using a 96-well broth microdilution assay according to Clinical and Laboratory Standards Institute (CLSI) guidelines^51^ in Sabouraud dextrose broth (SDB) against both a standard strain of *Candida albicans* (ATCC 90029) and a corresponding a local clinical isolate that was isolated from a burn patient at Burns Centre Emergency Hospital in Sulaimani city, and identified using VITIK-2. Inocula were prepared by adjusting freshly grown microbial cultures to a 0.5 McFarland standard (1 × 10^6^ CFU mL^−1^). In each well of a sterile 96-well plate, 100 µL of two-fold serial dilutions of VNPs at concentrations ranging from 4 µg mL^−1^ to 500 µg mL^−1^ was mixed with 100 µL of SDB containing the microbial suspension, resulting in a final volume of 200 µL per well. Control wells included media with microbes (positive control) and media with VNPs only (negative control). Plates were incubated at 35 °C for 24 h, after which optical density was measured at 600 nm using a microplate reader (Biotech µquant, USA). The minimum inhibitory concentration (MIC) was determined as the lowest VNP concentration that showed no visible microbial growth compared to the untreated control.

### 2.9 Biofilm disruption assay

The biofilm disruption activity of VNPs was evaluated against clinical biofilm-forming isolates of *Candida albicans* according to the method of Wang *et al.*^52^ Briefly, 200 µL of standardized microbial suspension (adjusted to 0.5 McFarland standard) was inoculated into sterile 96-well microtiter plates and incubated under static conditions for 48 h at 37 °C to allow biofilm formation. Following incubation, planktonic cells were gently removed, and wells were washed twice with sterile phosphate-buffered saline (PBS) to eliminate non-adherent cells. The preformed biofilms were then treated with VNPs at various concentrations, including MIC, ½ MIC, ¼ MIC, and ⅛ MIC, in a total volume of 200 µL per well. Control wells were left untreated, and all plates were incubated for an additional 24 h. Subsequently, the wells were washed with PBS, and the residual biofilm biomass was fixed and stained with 0.1% crystal violet for 15 min. Excess stain was removed, wells were rinsed with PBS, and bound dye was solubilized by adding 200 µL of 96% ethanol. The optical density (OD) was measured at 570 nm using a microplate reader (Biotech µquant, USA). The percentage reduction in biofilm biomass was calculated by comparing treated wells to untreated biofilm controls.

### 2.10 Anticancer activity (MTT assay)

The anticancer activity of VNPs was determined *in vitro* using the MTT [3-(4,5-dimethylthiazol-2-yl)-2,5-diphenyltetrazolium bromide] assay,^53^ employing Promega's CellTiter 96^®^ AQueous One Solution Cell Proliferation assay kit (#G4000), in two human pancreatic cancer cell lines, T3M-4 and CD18/HPAF that obtained from ATCC (Manassas, VA). Briefly, cell lines were plated in 96-well tissue culture plates at an appropriate density and incubated at 37 °C for 24 h in 5% CO_2_. Cells were then exposed to VNPs at serial dilutions ranging from 16 µg mL^−1^ to 512 µg mL^−1^. After 24 h of treatment, dose-dependent cell viability was assessed by optical OD measurement at 570 nm. A narrower concentration range (2, 4, and 8 µg mL^−1^) was subsequently employed for analysis of cytotoxicity over a further 24 h, confirming efficacy at lower doses.

For time-dependent analysis, cells were treated with the optimized concentrations for 5 days, and duplicate plates were read daily. Cell viability was assessed at 570 nm using a plate reader (BioTek Synergy H1, USA), and all treatments were performed in triplicate. Data were normalized to untreated controls, and statistical significance was determined at *p* < 0.05.

### 2.11 Colony formation assay

**Fig. 1:**
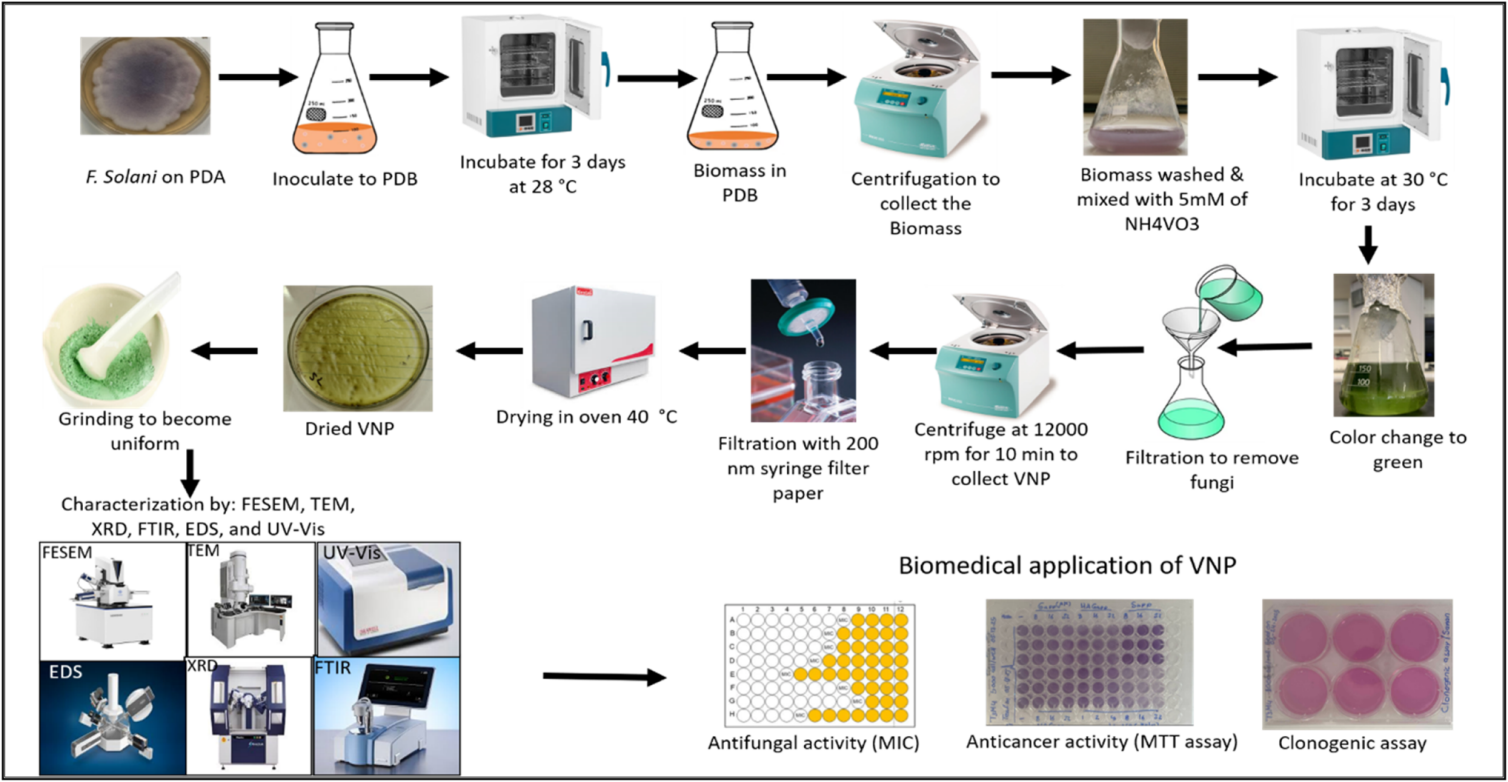
Schematic diagram of mycosynthesis of VNPs using *F. solani* and their bio applications.

The effect of VNPs on the clonogenic potential of pancreatic ductal adenocarcinoma cell lines (T3M-4 and CD18/HPAF) was evaluated using a colony formation assay according to the method of Rafehi *et al.*^54^ Briefly, cells were seeded into six-well plates at a density of 1000 cells/well in Dulbecco's Modified Eagle Medium (DMEM) supplemented with 7% fetal bovine serum (FBS), 1% l-glutamine, and 1% penicillin–streptomycin (PS), and incubated for 24 h under standard conditions (37 °C, 5% CO_2_). The medium was then replaced with fresh medium containing 2 µg mL^−1^ or 4 µg mL^−1^ of VNPs, while control wells received fresh medium without VNPs. Cells were further incubated for 12 days to allow colony formation. After incubation, the medium was aspirated, cells were washed twice with PBS (5 min per wash), fixed with 4% paraformaldehyde for 20 min at 4 °C, and washed again. After staining colonies with 0.5% crystal violet for 10–30 min at room temperature, they were rinsed with distilled water and air-dried. Colonies were counted manually under a stereomicroscope, and only clusters with more than 50 cells were designated as colonies. Fig. 1 illustrates the overall study design, encompassing VNP synthesis, characterization, and bioactivities.

### 2.12 Statistical analysis

GraphPad Prism version 10.1.0 (GraphPad Software, San Diego, CA, USA) was used to perform the statistical analyses. A two-way ANOVA was used to determine the significance between independent variables such as concentration and exposure time in the MTT experiment. A one-way ANOVA was performed to identify significant differences between the different treatment groups. Additionally, ImageJ software (version 1.54g, National Institutes of Health, Bethesda, MD, USA) was used to calculate the standard deviations and the diameters of the colony growth and particle size distributions. OriginPro 2024 (version 10.1.0.178, OriginLab Corporation, Northampton, MA, USA) was used to generate clear and accurate graphs. For all tests, *p* < 0.05 was used to indicate statistical significance.

## 3 Results

### 3.1 Vanadium tolerance of *Fusarium solani*

**Fig. 2:**
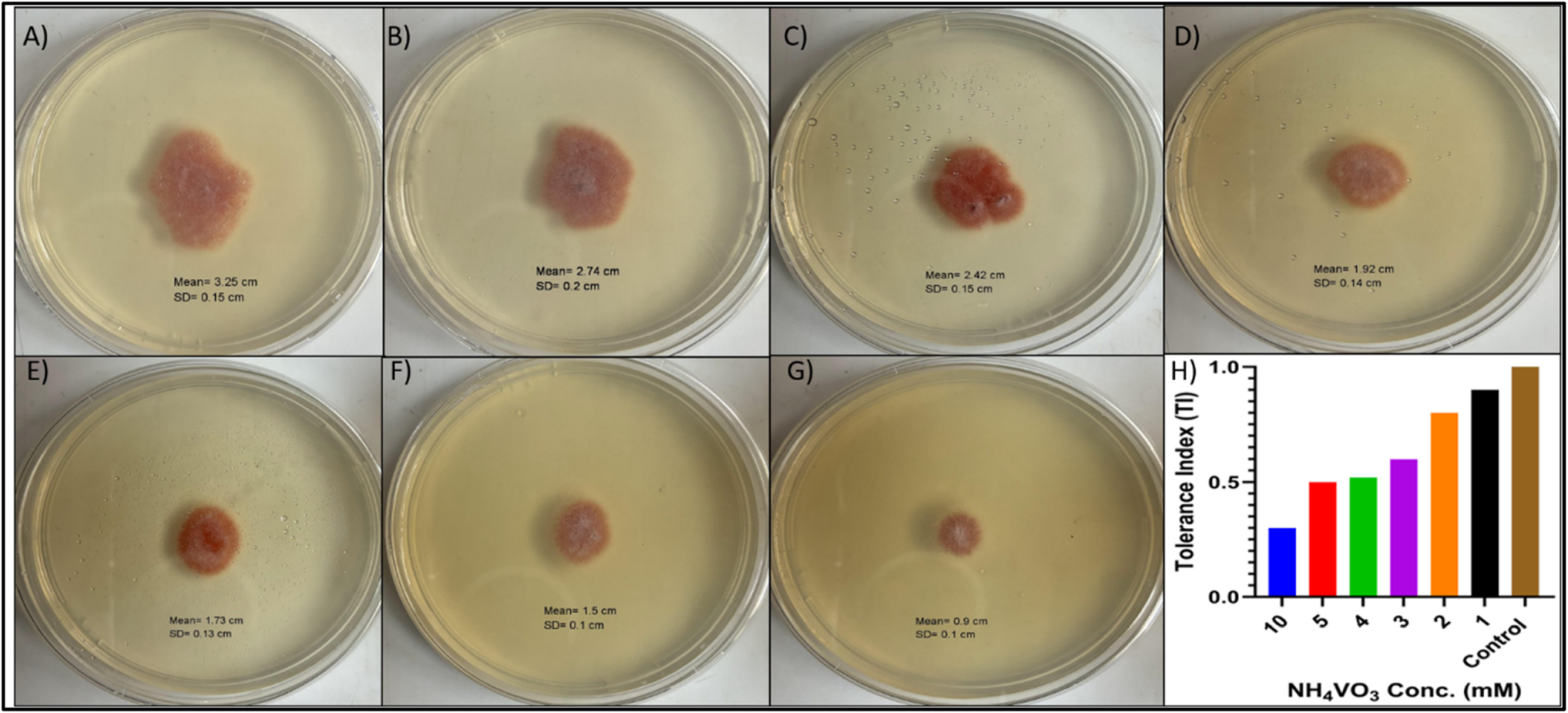
Effect of increasing ammonium metavanadate (NH_4_VO_3_) concentrations on fungal growth and tolerance. (A–G), Fungal colony growth after 3 days of incubation on PDA plates supplemented with NH_4_VO_3_ at the following concentrations: (A) control growth (0 mM), (B–G) 1, 2, 3, 4, 5, and 10 mM, respectively. Colony diameter decreases progressively with higher metal concentrations, indicating toxicity. (H) Corresponding Tolerance Index (TI) values. The TI decreases significantly as NH_4_VO_3_ concentration increases, highlighting the strain's sensitivity to vanadium stress, particularly at 10 mM.

The tolerance index (TI) assay revealed that for *F. solani*, the growth diameter of the fungus declined as concentration increased from 1–10 mM, indicating a clear dose-dependent inhibitory effect on fungal growth. The TI remained high (>0.5) at 1–5 mM, suggesting lower effects on fungal biomass. However, the greatest growth inhibition was noticed when the fungus was subjected to 10 mM of the NH_4_VO_3_, where the TI was <0.5. Notably, there was no enhancement of the fungal growth at any concentration used (Fig. 2).

### 3.2 Initial antimicrobial screening and optimization of synthesis conditions

The antifungal activity of the synthesized VNPs against *C. albicans* was evaluated in comparison to the precursor salt and fungal metabolites. The mycosynthesized VNPs exhibited antifungal activity, whereas the controls showed no effect (Fig. S1). Optimization was subsequently performed to identify the VNP variant with the highest bioactivity.

**Fig. 3:**
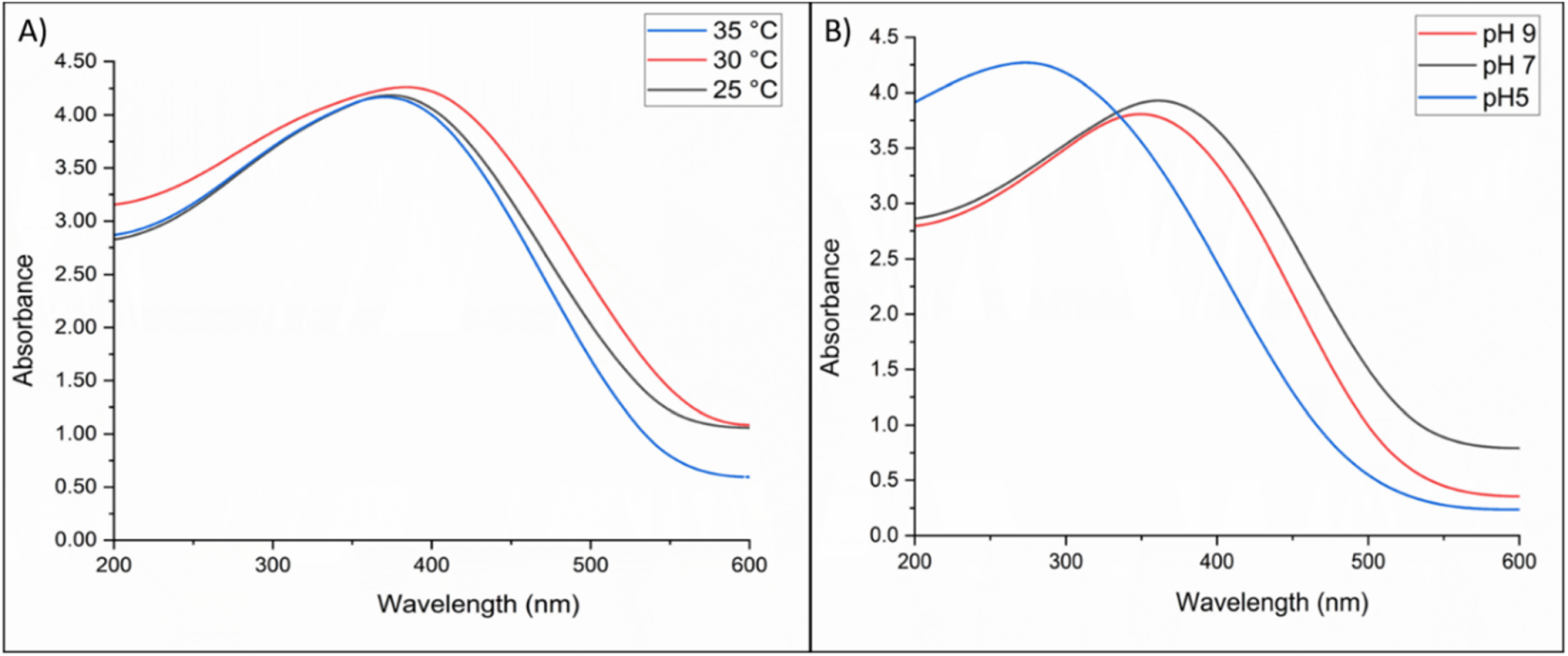
UV-vis absorption spectra of biosynthesized VNPs under varying synthesis conditions. (A) Absorption spectra at different temperatures (25 °C, 30 °C, and 35 °C). The highest absorbance (∼4.3 a.u.) was observed at 30 °C, indicating optimal nanoparticle formation at this temperature. (B) Absorption spectra at different pH levels (pH 5.0, 7.0, and 9.0). Maximum absorbance (∼4.2 a.u.) occurred at pH 5.0 followed by pH 7.0 and 9.0, demonstrating the significant influence of pH on VNP synthesis.

UV-vis spectroscopy was used to evaluate the absorption spectra of VNPs synthesized under different conditions (temperature and pH). Across the different temperatures used 25 °C, 30 °C, and 35 °C, all spectra showed an absorption peak around 370 nm. However, the highest intensity was recorded for the VNPs that were incubated at 30 °C, which was ∼4.3 a.u., followed by 35 °C and 25 °C (Fig. 3A), indicating the most favorable synthesis temperature. At pH 5.0, the synthesis showed the strongest absorbance (∼4.2 a.u.) with a broad peak at 280 nm, whereas pH 7.0 and 9.0 produced moderate peaks at 370 nm (Fig. 3B). Thus, 30 °C and pH 5.0 were optimal for yield. Contrastingly, regarding bioactivity, while all samples were effective against *C. albicans*, the VNPs synthesized at pH 7.0 and 30 °C demonstrated superior antifungal efficacy (Fig. S2).

### 3.3 Characterization of VNPs

VNPs characterization involves investigating their size, shape, and surface characteristics as well as their chemical and physical properties using a variety of methods, including:

#### 3.3.1 Ultraviolet-visible spectroscopy

**Fig. 4:**
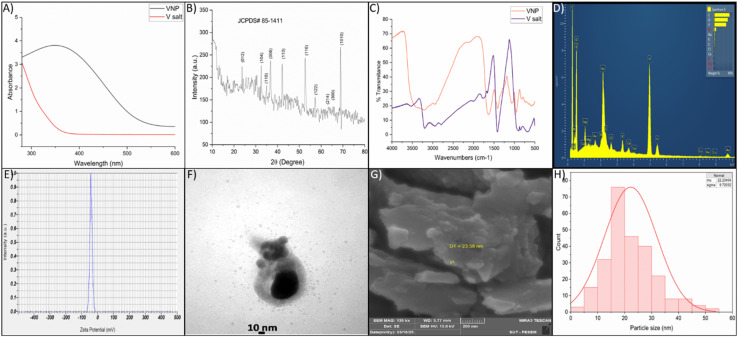
Characterization of *F. solani*-mediated VNPs. (A) UV-vis spectrum showing the distinct surface plasmon resonance (SPR) peak of biosynthesized VNPs compared with vanadium salt solution. (B) XRD pattern confirming the crystalline morphology of the nanoparticles. (C) FTIR spectra of VNPs and precursor with different functional groups involved in VNP formation. (D) EDX analysis confirming the elemental composition and purity of VNPs. (E) Zeta potential measurement indicating strong negative surface charge (−40.8 mV). (F) TEM image showing morphology with an average diameter of ∼10–30 nm. (G) SEM micrograph illustrating aggregated nanostructures with sizes ranging between 20–30 nm. (H) Particle size distribution histogram showing average particle size of 22 nm and SD ± 9 nm.

UV-visible spectroscopy was utilized to evaluate the optical properties of the biosynthesized VNPs. The VNP spectrum exhibited a clear surface plasmon resonance (SPR) band around 370 nm. The control solution containing solely the vanadium precursor exhibited minimal absorption in the visible spectrum. The observation of a distinct and well-defined SPR peak, coupled with the absence of broad background bands, indicates a narrow particle size distribution and limited agglomeration (Fig. 4A).

#### 3.3.2 XRD analysis

The XRD analysis revealed the crystalline characteristics of the VNPs, as evidenced by distinct peaks. The VNPs exhibited a rhombohedral structure, with the diffraction patterns displaying notable peaks at 2*θ* ≈ 24°, 33°, 36°, 38°, 21°, 53°, 57°, 64°, and 67° corresponding to crystallographic planes including *hkl* (012), (104), (110), (006), (113), (116), (122), (214) and (300), consistent with JCPDS card no. 85-1411. The Debye–Scherrer equation revealed that the crystal size of VNPs is approximately 24 nm, signifying their nanoscale dimensions (Fig. 4B).

#### 3.3.3 FTIR analysis

FTIR analysis shows the chemical components that were secreted by *F. solani* in the reduction and stabilization of VNPs. The FTIR spectrum presented in Fig. 4C illustrates a clear distinction between the precursor salt and VNPs, confirming chemical interactions during VNP synthesis. The precursor salt showed a weak peak around ∼3400 cm^−1^ and ∼1640 cm^−1^, which indicate the presence of O–H and H–O–H bending vibrations from the aqueous environment, without any significant organic molecules. In contrast, the VNP spectra showed different prominent peaks including: ∼3400 cm^−1^ (O–H/N–H stretching from phenolics, alcohols, and proteins), ∼2920 cm^−1^ (C–H stretching), ∼1650 cm^−1^ (amide I, C=O stretching), ∼1540 cm^−1^ (amide II, N–H bending/C–N stretching), ∼1400 cm^−1^ (–COO^−^ symmetric stretching), and ∼1000–1100 cm^−1^ (C–O stretching from polysaccharides).

There were also additional peaks between 500 and 700 cm^−1^ that matched V–O and V–O–V vibrations, which showed that vanadium oxide was forming. These spectra indicate that fungal biomolecules worked as reducing and capping agents for VNPs (Fig. 4C).

#### 3.3.4 Energy-dispersive X-ray spectroscopy (EDS)

The EDS spectra revealed prominent peaks for vanadium (V), oxygen (O), and carbon (C), thereby elucidating the composition of the synthesized nanoparticles. The distinct V signal confirmed the presence of the target element, while the oxygen signal indicated that the surface exhibited only partial oxidation, a feature commonly associated with nanoscale transition metals. The minor peaks observed for nitrogen, sodium, magnesium, phosphorus, sulfur, chlorine, potassium, and calcium are likely the result of residual precursors or ambient adsorption phenomena. The observed peaks for Au and Cu can be attributed to the SEM grid and the materials used for coating. The quantitative EDS analysis indicated that vanadium was the predominant element, thus validating the effective synthesis of the nanoparticles (Fig. 4D). The detailed elemental composition of the VNPs is shown in Table S1.

#### 3.3.5 Zeta potential analysis

The zeta potential analysis indicated an average value of −40.8 mV, alongside an electrophoretic mobility of −0.000315 cm^2^ V^−1^ s^−1^ at a temperature of 24.9 °C, in a medium defined by a viscosity of 0.897 mPa s^−1^ and a conductivity of 0.424 mS cm^−1^ (Fig. 4E). The negative zeta potential signifies the effective dispersion of the VNP colloidal solution.

#### 3.3.6 Morphological characteristics using (FESEM and TEM)

FESEM micrographs revealed irregularly shaped particles with noticeable aggregation.

Image analysis showed a polydisperse particle size distribution (Fig. 4G). This heterogeneity likely resulted from both primary particles and secondary agglomerates formed during synthesis or drying. High-resolution TEM provided detailed insights into nanoparticle morphology. TEM images at 10 nm scale displayed distinct, well-separated nanoparticles alongside small clusters. The predominant particle diameter was ∼24 nm, with smaller (<10 nm) particles dispersed around aggregates. The darker contrasts corresponded to crystalline cores of higher electron density, confirming their solid nature (Fig. 4F and G).

Together, FESEM and TEM analyses demonstrate that the VNPs are nanosized, polydisperse, and partially aggregated; characteristics that may influence surface reactivity and biological performance. The size distribution curve (Fig. 4H) showed that most nanoparticles sizes were ranged from 10–30 nm with average of 22 nm and standard deviation of ± 9 nm.

### 3.4 Antifungal activity of VNPs

**Fig. 5:**
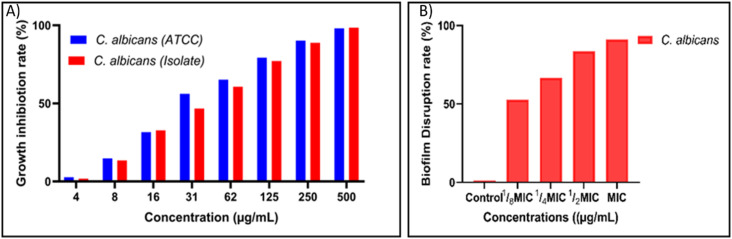
Antifungal and antibiofilm activities of biosynthesized VNPs against *Candida albicans*. (A) VNPs inhibited growth of both ATCC reference strain and multidrug-resistant clinical isolate in a concentration-dependent manner, with an MIC of 125 µg mL^−1^. (B) Biofilm disruption exceeded 80% at MIC and ½ MIC, remained partial at ¼ MIC, and was minimal at ⅛ MIC.

The antifungal potential of VNPs was evaluated against *Candida albicans* ATCC reference strain and a multidrug-resistant (MDR) clinical isolate. Growth inhibition assays revealed a concentration-dependent suppression for both strains. The minimum inhibitory concentration (MIC), defined as the lowest concentration showing no visible growth, was 125 µg mL^−1^ for both strains. Although the MDR strain exhibited slightly reduced inhibition at intermediate concentrations, the MIC values were identical, indicating comparable susceptibility (Fig. 5A).

### 3.5 Biofilm disruption assay

Biofilm disruption was examined at MIC, ½ MIC, ¼ MIC, and ⅛ MIC concentrations.

For *C. albicans*, biofilm biomass decreased by more than 80% at MIC and ½ MIC for the clinical isolate, while ¼ MIC still produced notable disruption and ⅛ MIC showed minimal effects (Fig. 5B). These observations highlight the strong antibiofilm activity of VNPs even at subinhibitory concentrations.

### 3.6 Cytotoxicity assessment (MTT assay)

**Fig. 6:**
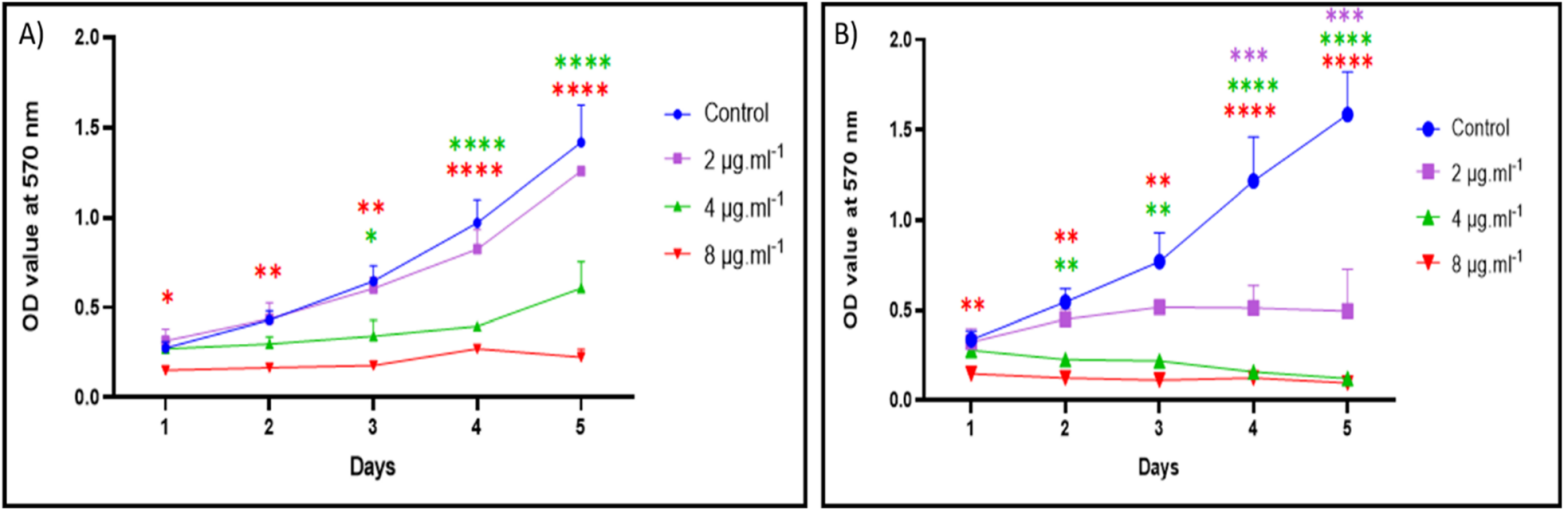
Time- and dose-dependent cytotoxic effects of VNPs on T3M-4 (A) and CD18/HPAF (B) pancreatic cancer cells assessed *via* MTT assay. Cells were treated with increasing concentrations of VNPs (2, 4, and 8 µg mL^−1^) over five days. Results are presented as mean ± SD from three independent experiments, and statistical significance was determined using two-way ANOVA.

The cytotoxic activity of VNPs was investigated against pancreatic cancer cell lines (T3M-4 and CD18/HPAF) using the MTT assay over five days (Fig. 6). Initially, the cytotoxic activity of the VNPs was evaluated using concentrations ranging from 16 to 512 µg mL^−1^. All tested doses exhibited significant cytotoxicity compared to the control (Fig. S3). Consequently, lower concentrations were employed to determine the half-maximal inhibitory concentration (IC_50_). A clear dose- and time-dependent reduction in cell viability was observed compared to untreated controls. Control cells displayed normal proliferation, reaching OD values of 1.8–2.0 at 570 nm by day 5. In contrast, VNP-treated cells exhibited progressively reduced metabolic activity as nanoparticle concentration increased. At 2 µg mL^−1^, a moderate inhibitory effect was observed; at 4 µg mL^−1^, a significant reduction (*p* < 0.01) occurred from day 2 onward; and at 8 µg mL^−1^, cell viability was markedly suppressed (*p* < 0.001) throughout the incubation period. These findings confirm strong, concentration-dependent antiproliferative effects of VNPs against pancreatic cancer cells.

### 3.7 Colony formation assay

**Fig. 7:**
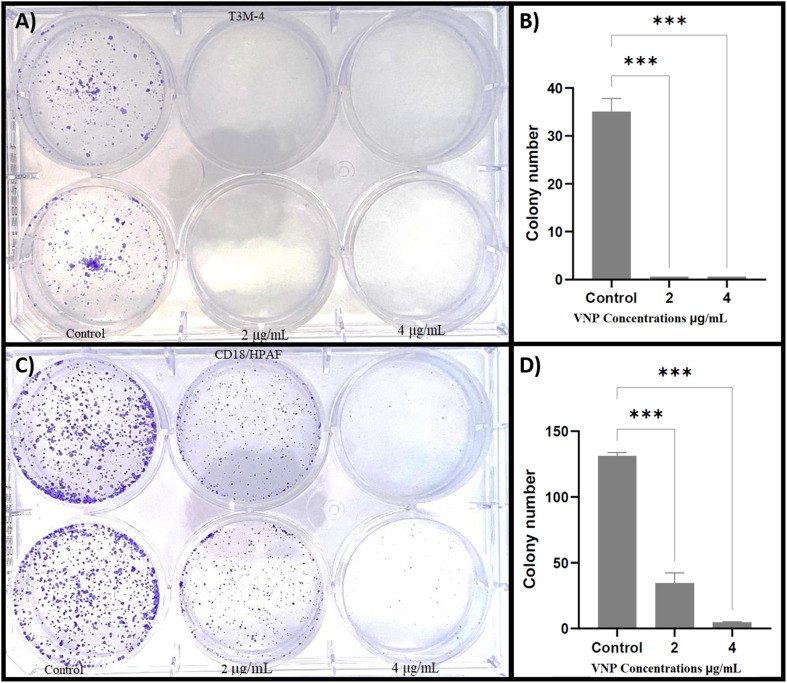
Dose-dependent suppression of clonogenic potential by VNPs in pancreatic cancer cell lines. Treatment with 2 µg mL^−1^ and 4 µg mL^−1^ of VNPs resulted in significant reductions in colony formation for both T3M-4 and CD18/HPAF cell lines (A and C) compared to untreated controls. T3M-4 cells were particularly sensitive, with colony formation almost entirely abolished at both concentrations. CD18/HPAF cells also showed a significant reduction in clonogenic survival (B and D). Quantitative analysis and statistical evaluation using one-way ANOVA.

The long-term cytotoxic potential of VNPs was further evaluated *via* clonogenic assay. CD18/HPAF and T3M-4 cells were treated with 0, 2, and 4 µg mL^−1^ VNPs and incubated for 12 days. Untreated controls formed an average of 131 colonies (CD18/HPAF) and 35 colonies (T3M-4). Treatment with 2 µg mL^−1^ reduced colony numbers to 34 and 4, respectively, while 4 µg mL^−1^ completely abolished colony formation in T3M-4 cells. These results demonstrate a pronounced, dose-dependent suppression of clonogenic potential (Fig. 7).

## 4 Discussion

To our knowledge, this is the first study investigating the use of *F. solani* for synthesizing vanadium nanoparticles. These findings support the hypothesis that *F. solani* can effectively drive VNP formation with efficacy comparable to or greater than other bio-methods. As a primary focus of this work, we demonstrated successful antigrowth and antibiofilm activities of VNPs against *Candida albicans*.

### 4.1 Tolerance index and synthesis optimizations

The Tolerance Index (TI) analysis revealed that *F. solani* growth was progressively inhibited by increasing concentrations of ammonium metavanadate (NH_4_VO_3_), demonstrating clear dose-dependent suppression as reported by Xu *et al.*^55^ Although the fungus exhibited partial tolerance at lower concentrations (1–5 mM) with TI values exceeding 0.5, a marked decline in growth was observed at 10 mM (TI < 0.2), suggesting pronounced toxicity and impaired hyphal development as reported by Ceci *et al.*,^56^ who found soil fungi became sensitive above 6 mM vanadium. This pattern is consistent with typical heavy-metal stress responses in fungi, wherein tolerance decreases proportionally with rising metal concentrations due to enzyme disruption and metal-binding at the cell wall.^57^ In particular, vanadium toxicity has been shown to induce morphological changes including thinner hyphae and damaged cell structures, resulting in reduced fungal biomass and diminished sporulation.^55,56^ Notably, TI values did not exceed 1.0 at any tested concentration, indicating that *F. solani* failed to develop adaptive tolerance or exhibit growth enhancement in response to vanadium exposure. This observation aligns with previous findings by Ceci *et al.*^56^ which suggest that rapid initial growth followed by high mortality limits fungal tolerance to metal stress. In summary, *F. solani* displays moderate tolerance to low vanadium concentrations but experiences significant growth inhibition at higher doses, a pattern consistent with typical responses of non-adapted soil fungi to heavy-metal stress.

### 4.2 Optimization of VNPs synthesis conditions

UV-visible spectroscopy confirmed the successful biosynthesis of VNPs by *F. solani* under various physicochemical conditions of pH and temperature, indicating the surface plasmon resonance (SPR) characteristic of vanadium oxide nanoparticles. Variations in absorbance intensities reflect the influence of environmental physicochemical parameters on nanoparticle formation. At pH 5.0, the formation of heterogeneous NPs was indicated by a broad peak shifting from 370 nm to 280 nm. Rajput *et al.*^16^ reported that acidic conditions often alter NP size and shape, leading to such hypsochronic shifts.^58,59^ The broader spectra observed at pH 5.0 likely result from simultaneous nucleation and slower reaction kinetics; these conditions favor particle growth and aggregation, thereby broadening the absorption band, as observed in our data^60–62^

In this study, the highest absorbance was achieved at acidic pH 5.0. This may be due to acidic conditions facilitating the transformation of vanadium species into vanadium oxide clusters, which then develop into isolated tetrahedral vanadium species. These species co-condense to form the final structure, as indicated by the broad UV-vis peak around 280 nm corresponding to charge-transfer transitions between oxygen ligands and tetrahedrally coordinated vanadium ions. The increased intensity of this band at lower pH suggests a higher abundance of isolated V species^63^ In contrast, neutral conditions (pH 7.0) yielded moderate absorbance, while alkaline environments (pH 9.0) showed reduced absorbance, likely due to biomolecular disruption or enzymatic deactivation^64,65^ Previous studies have demonstrated that neutral to slightly basic pH favors the formation of stable, spherical nanoparticles, whereas acidic environments promote irregular shapes. Additionally, particle size decreases with increasing alkalinity, thereby enhancing mono-dispersity, as observed in this study.^66,67^ Based on these findings, we hypothesize that increasing pH enhances the reduction potential of vanadium because fungal metabolites become more reactive and ionized, resulting in more uniform and stable nanoparticles. Conversely, at acidic pH, these metabolites become protonated and less active, which may affect the nanoparticle absorption spectra.^68–71^

Regarding the temperature, the results showed that at temperatures of 25 °C, 30 °C, and 35 °C, VNPs synthesis exhibited similar spectral profiles, with the highest peak absorbance observed at 30 °C, and weaker absorbance at 35 °C, indicating optimal production conditions, which aligns with Kumari *et al.*,^72^ whose study showed that NP biosynthesis at high temperatures yields larger NPs regardless of pH. In this study, the most favorable temperature with suitable antimicrobial activity was 30 °C, as reported by Kumari *et al.*,^65^ who found that the optimal temperature for biosynthesis of NPs is 30 °C to form small and tunable size to be effective against MDR. These observations align with earlier studies demonstrating that moderate temperatures favor the formation of smaller, more uniform nanoparticles, whereas higher temperatures or prolonged incubation produce larger, irregular forms.^65^ The moderately reduced intensity at 25 °C likely stems from slower enzymatic activity, while the diminished absorbance at 35 °C may result from partial enzyme denaturation or nanoparticle aggregation, both of which impede nanoparticle synthesis. These findings are further supported by earlier studies establishing that moderate temperatures promote effective bio-reduction and formation of well-dispersed metal nanoparticles.^50,73^

### 4.3 Characterization of biosynthesized VNPs

#### 4.3.1 UV-visible spectroscopy

The characteristic green coloration observed during synthesis serves as a visual indicator of VNP (V_2_O_3_) formation.^74^ The control spectrum displayed baseline absorbance for the vanadium salt solution, whereas the biosynthesized VNP sample exhibited a distinct SPR peak at approximately 370 nm, confirming nanoparticle formation. The same spectra were observed for vanadium NPs in previous studies.^75,76^

#### 4.3.2 X-ray diffraction (XRD) analysis

The X-ray diffraction (XRD) pattern of the synthesized V_2_O_3_ nanoparticles displays a series of well-defined, sharp peaks distributed from 2*θ* ≈ 20° to 70°, confirming the crystalline nature of the sample. These peaks correspond to the rhombohedral structure of vanadium(iii) oxide (V_2_O_3_),^77^ in agreement with standard JCPDS data (card no. 85-1411) data file; *R*3̄*c* space group. The most intense diffraction peaks appear near 2*θ* ≈ 24°, 33°, 36°, 38°, 41°, 53°, 57°, 64°, 67° and 70°, corresponding to crystallographic planes (*hkl*), denoted by the Miller indices (012), (104), (110), (006), (113), (116), (122), (214), (300) and (1010).^78–80^ A strong signal observed at 24.0° is attributed to the (012) lattice plane, which is the signature for basal spacing (∼0.36 nm) and the rhombohedral structure of vanadium(iii) oxide (V_2_O_3_). The reflection at 33°, indexed to the (104) plane, serves as a fingerprint for the V_2_O_3_ structure. Furthermore, *c*-axis stacking and in-plane symmetry were confirmed by the peaks at 36° and 38°, representing the (110) and (006) planes, respectively. The (113) reflection at 41° provides insights into oxygen sublattice ordering and lattice distortions. Finally, high-angle peaks at 53°, 57°, and 64° attributed to (116), (122), and (214) imply exceptional crystallinity, while the (300) and (1010) reflections at 67° and 70° further support the formation of a highly organized, stoichiometric V_2_O_3_ phase.^81–83^

The sharpness and high intensity of these peaks indicate the formation of highly crystalline nanoparticles, while the absence of peak broadening suggests relatively larger crystallite dimensions and well-developed structural order. Collectively, the XRD analysis reveals that fungal metabolites facilitated the synthesis of V_2_O_3_ nanoparticles, likely *via* the bioreduction to form V(iii). However, further confirmation using X-ray Photoelectron Spectroscopy (XPS) is needed to conclusively determine the oxidation states.

#### 4.3.3 Fourier-transform infrared (FTIR) spectroscopy

Following *F. solani*-mediated synthesis, the biosynthesized VNPs and the vanadium salt exhibit a distinct biochemical transition, according to the FTIR comparison. While the VNP spectra showed several broad bands attributed to O–H, N–H, amide, carboxylate, and C–O vibrations from fungal metabolites as reported by Rajakumar *et al.*,^84^ indicating that fungal peptides are present in the biosynthesis of NPs, the salt spectrum only showed minor water-related peaks (∼3400 and 1630 cm^−1^), confirming its inorganic character. Strong absorption peaks between 500 and 700 cm^−1^, which were attributed to V–O and V–O–V bonds, was reported to confirm that VNPs were formed,^77^ while the absence of these peaks was observed in the precursor salt. Collectively, these spectral changes demonstrate the role of fungal metabolites in reducing vanadium ions and capping nanoparticles, thereby facilitating the formation of bio-functionalized, oxide-based VNPs.

#### 4.3.4 Energy-dispersive X-ray spectroscopy

The energy-dispersive X-ray spectroscopy spectrum of the biosynthesized VNPs reveals the elemental composition and validates the successful synthesis of vanadium-based nanomaterials. Strong and well-defined peaks for vanadium (V) and oxygen (O) dominate the spectrum, confirming the formation of vanadium oxide nanoparticles.^85^ The high intensity of these peaks indicates that vanadium constitutes the primary constituent, and the concurrent detection of oxygen supports the oxide stoichiometry. The detection of carbon (C), nitrogen (N), and sulfur (S) is attributed to organic biomolecules from *F. solani*, which function as reducing and stabilizing agents during synthesis. Minor elements including sodium (Na), potassium (K), magnesium (Mg), calcium (Ca), and phosphorus (P) are also present and likely originate from the fungal culture medium or residual cellular components.^86^ Additionally, peaks corresponding to gold (Au) and copper (Cu) are attributed to the coating or sample grid used during SEM-EDS sample preparation.

#### 4.3.5 Morphological characterization (SEM and TEM)

The morphological characteristics of the biosynthesized VNPs were examined using both SEM and TEM. The SEM images reveal that the nanoparticles exhibit a rough and irregular surface morphology with notable aggregation. Particles appear clustered, likely due to drying effects or residual bio-organic compounds from the fungal extract. Nevertheless, distinct nanostructured domains are evident, confirming nanoscale particle formation. In contrast, the TEM images provide substantially higher resolution and clearly demonstrate individual nanoparticles relatively spherical to slightly irregular morphologies with relatively smooth surfaces. The observed particle sizes fall well within the nanometer range, confirming effective size control during biosynthesis. Notably, the uniformity in particle contrast and boundaries indicates good crystallinity and phase homogeneity, which corroborates the XRD findings. The darker contrast observed in the central region of particles suggests a denser core, likely comprising crystalline vanadium oxide, while the lighter periphery may represent organic capping layers contributed by fungal metabolites. Collectively, SEM and TEM analyses confirm that *F. solani* effectively facilitates the formation of well-defined, crystalline, nanoscale VNPs, with organic biomolecules contributing to particle morphology and surface stabilization.

The particle size distribution histogram reveals a broad range spanning approximately 5 nm to 55 nm, with the majority of particles concentrated in the 10–30 nm range with average 22 nm SD ± 9 nm and a modal peak at 15–20 nm. This distribution profile confirms the nanoscale nature of the particles, consistent with TEM and XRD observations. The modest polydispersity observed may result from biological synthesis conditions, wherein multiple biomolecules simultaneously influence nucleation and particle growth.

#### 4.3.6 Zeta potential analysis

The zeta potential of the biosynthesized VNPs was measured at approximately −40.8 mV, indicating a highly electrostatically stabilized VNP.^30^ This pronounced negative surface charge implies substantial electrostatic repulsion between particles, facilitating short-term stability of the aqueous dispersion. The same result was reported by El-Naggar *et al.*,^87^ who showed that biosynthesis in neutral to basic solutions will form stable, negatively charged NPs. This stability results from the adsorption of oxygen-containing functional groups and potential surface oxidation of vanadium, making VNPs particularly favorable for biological applications.^88^ This zeta potential value is typically attributed to the presence of negatively charged functional groups, such as carboxylates and hydroxyls, from fungal metabolites secreted by *F. solani*, which function as natural capping and stabilizing agents, as determined by Gholami-Shabani.^43^

### 4.4 Antifungal activity of VNPs

The present findings demonstrate concentration-dependent antifungal activity of VNPs against *Candida albicans*, including both ATCC reference strain and clinical isolates, which support other research findings that VNPs have potential antifungal activity.^43,89–91^ Complete growth inhibition was achieved at 125 µg mL^−1^, thereby validating the fungicidal potential of VNPs, which likely operate through multiple mechanisms: reactive oxygen species (ROS) formation, disruption of membrane ergosterol, and mitochondrial damage.^89,92^ Notably, the demonstrated efficacy against clinical strains illustrates that VNPs remain effective despite phenotypic diversity and potential antifungal resistance, positioning them as promising alternatives to standard antifungal therapeutics.

### 4.5 Biofilm disruption assay

Biofilm disruption assays demonstrated that VNPs achieve significant biofilm reduction in *C. albicans* isolates. Maximum biofilm disruption (80–100%) was observed at MIC and ½ MIC concentrations, with disruption progressing in a dose-dependent manner. Similar results were reported by Silva *et al.*,^89^ and Natalio *et al.*^93^ The ability of VNPs to disrupt mature biofilms reflects their nano-bio interface properties, potentially including penetration into the extracellular polymeric matrix, oxidative damage induction, and metabolic interference, such as quorum-sensing disruption, to inhibit biofilm formation.^94^ The exact mechanisms underlying VNP-mediated biofilm disruption in *C. albicans* remain incompletely understood and warrant further investigation. However, nanoparticle antifungal activity is regulated by multiple factors, including composition, size, surface charge, and geometric shape, in which triangular-shaped nanoparticles demonstrate greater toxicity compared to spherical or rod-shaped alternatives.^95–99^ Potential mechanisms of biofilm disruption include physical disintegration of biofilm architecture, suppression or destruction of extracellular polymeric substances (EPS), disruption of quorum-sensing pathways, and inhibition of biofilm-associated virulence factors.^100–102^ These results are particularly significant given the inherent resistance of biofilm-encased cells to conventional antifungals and the potential applications of VNPs for medical device coatings and other antimicrobial surfaces.

### 4.6 Anticancer activity of VNPs

Although this study was not designed to assess the mode of action against human cell lines, these results are promising and warrant further investigation. Future studies should assess VNPs' mechanisms of action across a variety of cell lines and establish clinically feasible production methods for testing in rodent and small animal models to translate these findings toward clinical applications. Pancreatic cancer represents a leading cause of cancer-related mortality globally. In 2012, approximately 338 000 individuals were diagnosed with pancreatic cancer worldwide, establishing it as one of the most prevalent malignancies. Despite significant advances in oncology, the estimated 5 year survival rate remains below 5%, underscoring its extremely poor prognosis.^103,104^ The present data conclusively demonstrate that VNPs exhibit strong anticancer activity against both T3M-4 and CD18/HPAF pancreatic cancer cell lines with an IC_50_ of ∼4 µg mL^−1^. VNPs have also been studied to have anticancer activity against different cell lines.^42,105,106^ Cell viability was reduced in a concentration- and time-dependent manner, indicating potent cytotoxicity, as reported by Nie *et al.*^44^ The toxicity of metallic nanoparticles is closely correlated with their size.^107,108^ Smaller nanoparticles generally produce elevated levels of reactive oxygen species (ROS),^109^ affect enzymatic activity, and substantially reduce cell viability compared to larger counterparts.^110,111^ This enhanced toxicity results from changes in surface properties and associated behaviors, including diffusion, attachment, and sedimentation, that accompany size variation. At the highest VNP concentration (8 µg mL^−1^), metabolic activity was substantially suppressed, likely through induction of oxidative stress, mitochondrial dysfunction, and apoptotic cell death.^112^

### 4.7 Differential sensitivity between cell lines

Although both cell lines demonstrated responsiveness to VNP exposure, T3M-4 cells exhibited greater sensitivity, particularly at moderate VNP concentrations (4 µg mL^−1^), suggesting potential differences in nanoparticle uptake, antioxidant capacity, and metabolic rates between cell lines.^113^ Sustained growth suppression with extended incubation periods further indicates that VNPs impair long-term proliferative capacity, potentially resulting from prolonged nanoparticle exposure rather than acute cytotoxic effects.^113^ This capacity for sustained growth inhibition underscores the considerable potential of VNPs for nanotherapeutic applications targeting pancreatic cancer, a malignancy characterized by poor prognosis and widespread chemoresistance.

### 4.8 Colony formation assay

The colony-forming (clonogenic) assay measures long-term reproductive survival rather than short-term metabolic activity and is therefore particularly informative for evaluating whether a treatment permanently disables a cell's ability to proliferate.^114,115^ As highlighted by Rundén-Pran *et al.*,^116^ the colony-forming efficiency (CFE) assay is particularly valuable for evaluating nanomaterial toxicity because it avoids false readings that can occur in colorimetric assays like MTT, which may interact with reactive nanoparticles. Hence, the marked reduction in CFE observed here represents genuine cytotoxic and cytostatic effects of VNP exposure.

In this study, treatment with VNPs caused a pronounced, dose-dependent decline in the number of colonies formed, reflecting strong inhibition of cellular proliferation and survival. According to Thapliyal *et al.*,^113^ the inability of cells to form colonies indicates a loss of reproductive potential, often resulting from DNA damage, apoptosis, or permanent metabolic arrest. The near absence of colonies at higher VNP concentrations therefore demonstrates that the nanoparticles not only suppress short-term metabolic activity but also permanently impair the cells' ability to replicate and form new populations. The observed decline in colony formation can be attributed to multiple, interrelated mechanisms of nanotoxicity. As Gutiérrez *et al.*,^117^ reported, nanoparticles frequently induce oxidative stress, mitochondrial damage, and DNA strand breaks, leading to apoptosis and growth inhibition. Vanadium-based nanostructures, in particular, undergo redox cycling between oxidation states (VO_*X*_), generating reactive oxygen species that disrupt vital cellular processes.

The inhibitory effect was statistically significant in both cell lines, reinforcing that the antiproliferative impact of VNPs is not restricted to one cancer cell type. The sustained growth suppression with prolonged incubation also implies that VNPs can disable long-term proliferative capability.^113^ Clonogenic inhibition is especially significant because it reflects the long-term ability of tumor cells to continue proliferating after treatment. Thus, the near-total disappearance of colonies at 4 µg mL^−1^ suggests that VNPs not only reduce metabolic activity but also eliminate the cells' capacity to recover and form new colonies, a key trait of successful anticancer agents. These results underscore the strong antiproliferative effect of biogenically synthesized VNPs and reinforce their potential as promising therapeutic candidates for pancreatic cancer, one of the malignancies with the poorest survival rates and high rates of chemoresistance.

## 5 Conclusion

This study successfully demonstrates an eco-friendly approach for the biosynthesis of VNPs, utilizing *F. solani* as a natural bio-factories by using fungal metabolites as agents for nanoparticle synthesis. During the optimization study, the results revealed that the optimal pH and temperature for VNP synthesis were neutral pH 7.0 and a temperature of 30 °C for antifungal activity, which notably improved both the yield and efficacy of the VNPs. Comprehensive spectroscopic and structural analyses using FTIR, XRD, FESEM, and TEM confirmed the production of stable, crystalline vanadium oxide nanoparticles, effectively capped with bioactive fungal metabolites. Functionally, the biosynthesized VNPs demonstrated robust antimicrobial activity against *C. albicans*, as well as notable cytotoxic effects against human pancreatic cancer cell lines. The observed IC_50_ value of approximately 4 µg mL^−1^ indicates significant antiproliferative potential, reinforcing the promise of VNPs as a novel candidate for anticancer therapeutics.

Overall, the integration of fungal biotechnology with nanoscience aligns closely with the principles of green chemistry, while offering a pathway toward the scalable production of multifunctional nanomaterials. This study underscores the potential of *F. solani*-mediated nanotechnology as a viable and sustainable platform for the development of next-generation therapeutic agents.

## Author contributions

This work is part of doctoral dissertation of Saman Mohammed under supervision of Prof. Dr Haider Hamzah. Saman carried out experimental design, synthesis, characterization, and experiments, data analysis, and draft preparation. Dr Hamzah provided guidance, critical review, supervision, editing and approval of final draft.

## Conflicts of interest

There are no conflicts to declare.

## Supplementary Material

RA-016-D5RA08636A-s001

## Data Availability

All data generated and analyzed in this study are included in the submitted article and supplementary information (SI). No external data were used. All relevant data are included; additional datasets can be provided by corresponding author. Supplementary information is available. See DOI: https://doi.org/10.1039/d5ra08636a.
