# Supplementary material for: Eco-friendly fabrication of vanadium nanoparticles via Fusarium solani with dual antifungal and anticancer bioactivities
Source: RSC Adv. 2026 Jan 8;16(3):2190–204. doi: 10.1039/d5ra08636a (PMC12780841; doi:10.1039/d5ra08636a)
Supplement: RA-016-D5RA08636A-s001 [file RA-016-D5RA08636A-s001.pdf]

Supplementary Information (SI) for RSC Advances.
This journal is © The Royal Society of Chemistry 2025

Supplementary

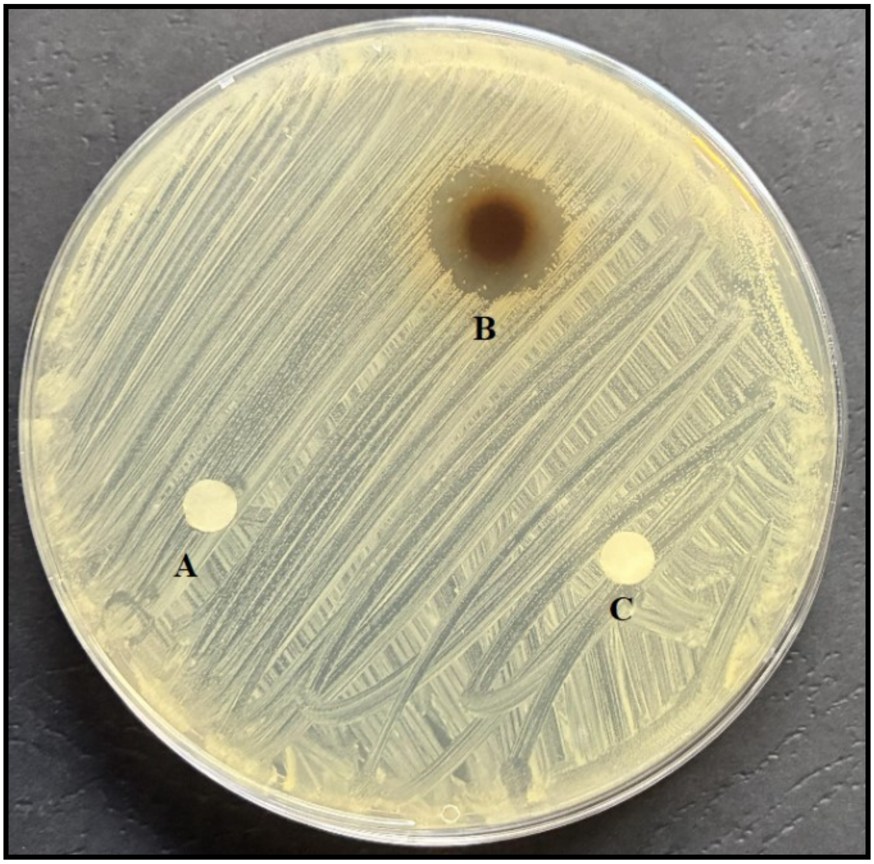

Fig. S1 initial antifungal screening for VNP against C. albicans. A) Fungal Extract showed no clear zone. B) VNP showed clear zone against C. albicans. C) ammonium metavanadate precursor without any zone of inhibition.

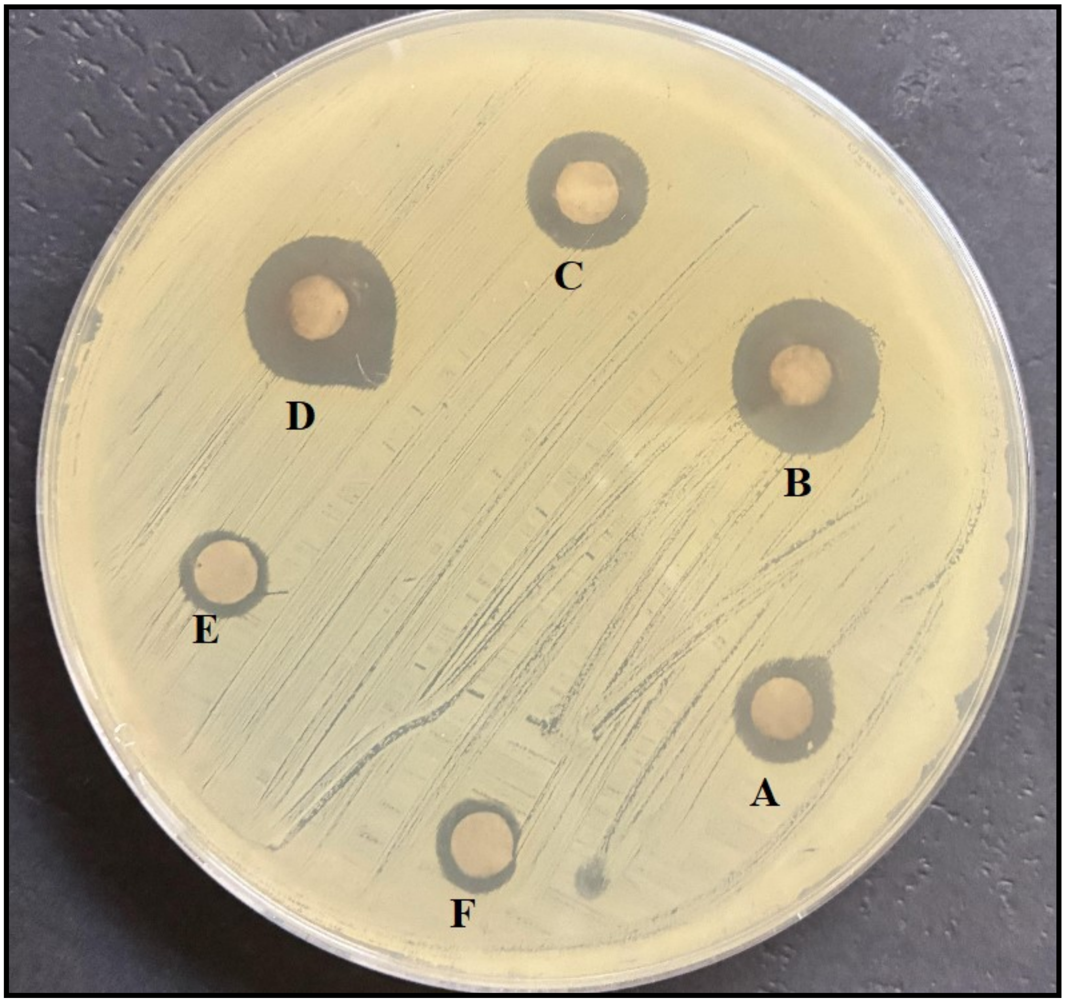

Fig. S2 optimization of VNP synthesis. Different pH and temperature for synthesis used. A) Temperature 25 °C, B) 30 °C. C) 35 °C. D) pH 7. E) pH 9. F) pH 5 used during synthesis. Temperature 30 °C and pH 7 showed most significant antifungal activity.

Table S1. EDS spectrum of biosynthesized V_2O_3 nanoparticles showing the elemental composition. Peaks corresponding to vanadium (V), carbon (C) and oxygen (O) confirm the NP core composition and biogenic synthesis using fungal metabolites as strong caping agent. Minor signals (Na, Mg, P, S, Cl, and K) may originate from fungal growth medium also their presence support biosynthesis and without altering V-base phase. (Cu) likely from grid.

Element	Apparent Concentration	k Ratio	Wt%	Wt% Sigma	Atomic %
C	1.63	0.01629	33.34	0.66	48.95
N	0.65	0.00117	4.74	1.29	5.97
O	1.93	0.00651	29.48	0.69	32.49
Na	0.24	0.00103	1.55	0.08	1.19
Mg	0.02	0.00013	0.15	0.05	0.11
P	0.10	0.00054	0.42	0.08	0.24
S	0.18	0.00152	1.18	0.09	0.65
Cl	0.11	0.00092	0.71	0.06	0.35
K	0.24	0.00203	1.47	0.07	0.66
Ca	0.11	0.00102	0.71	0.07	0.31
V	3.30	0.03297	26.20	0.48	9.07
Cu	0.00	0.00004	0.07	0.17	0.02
Total:			100.00		100.00

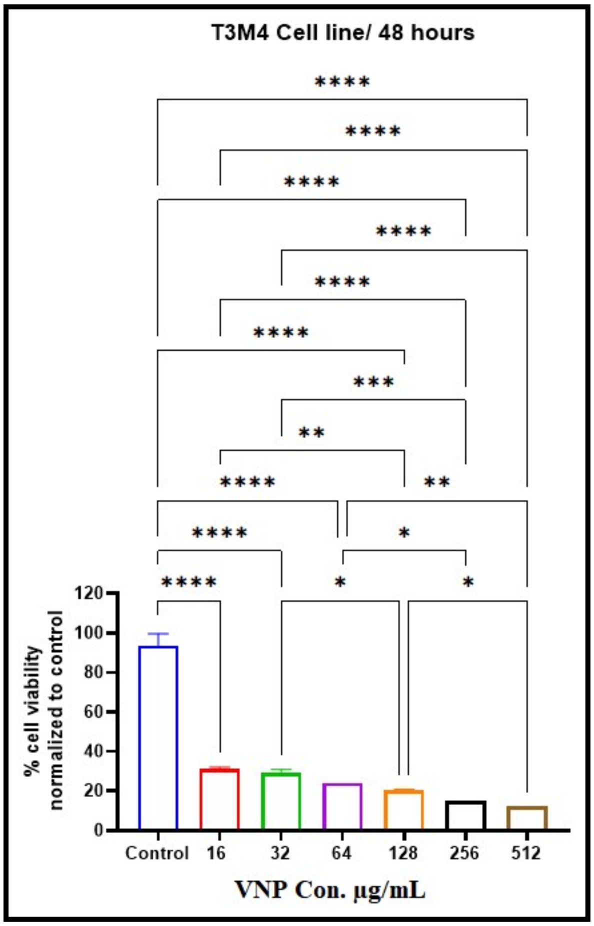

Fig. S3 Initial anticancer activity for VNP. High concentrations were used to determine the anticancer activity. All concentrations were showed significant anticancer activity. The doses were reduced to determine the IC50
